# Supplementary material for: Mechanically Strong and Tailorable Polyimide Aerogels Prepared with Novel Silicone Polymer Crosslinkers
Source: Gels. 2022 Jan 12;8(1):57. doi: 10.3390/gels8010057 (PMC8774617; doi:10.3390/gels8010057)
Supplement: Supplementary file 1 [file gels-08-00057-s001.zip › gels-1505816-supplementary.pdf]

gels

Article

Mechanically Strong and Tailorable Polyimide Aerogels Prepared with Novel Silicone Polymer Crosslinkers

Zhongxin Zhang [1], Yurui Deng [1], Zhiyi Lun [1], Xiao Zhang [1,2], Mingyuan Yan [1], Pan He [1], Caihong Li [1] and Yuelei Pan [1,*]

Supplementary Materials

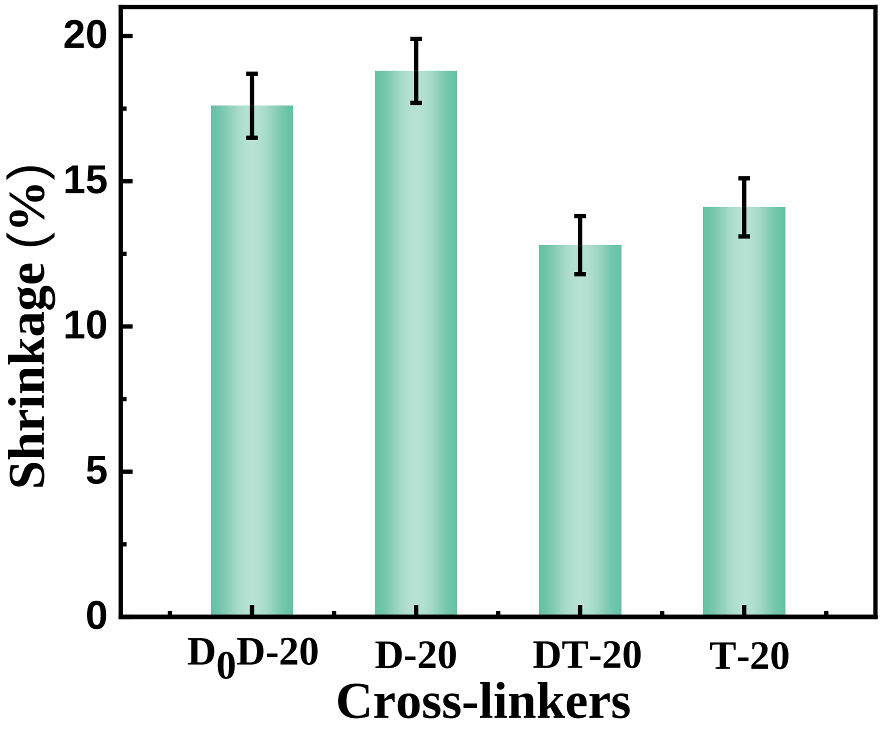

Figure S1. The shrinkage of aerogels cross-linked with silicone polymers prepared from different precursors.

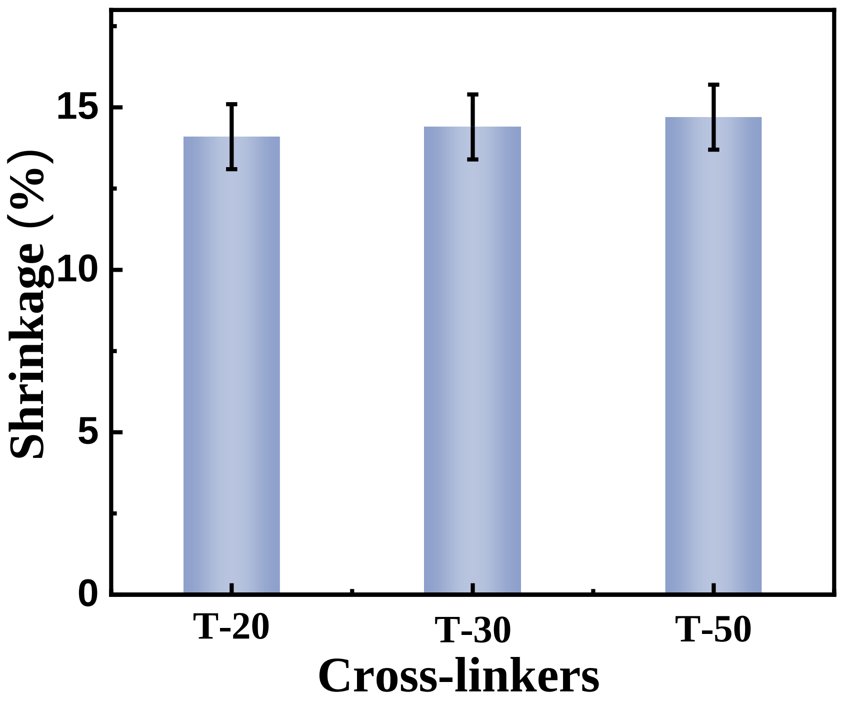

Figure S2. The shrinkage of aerogels prepared with silicone polymers of different degrees of polymerisation.

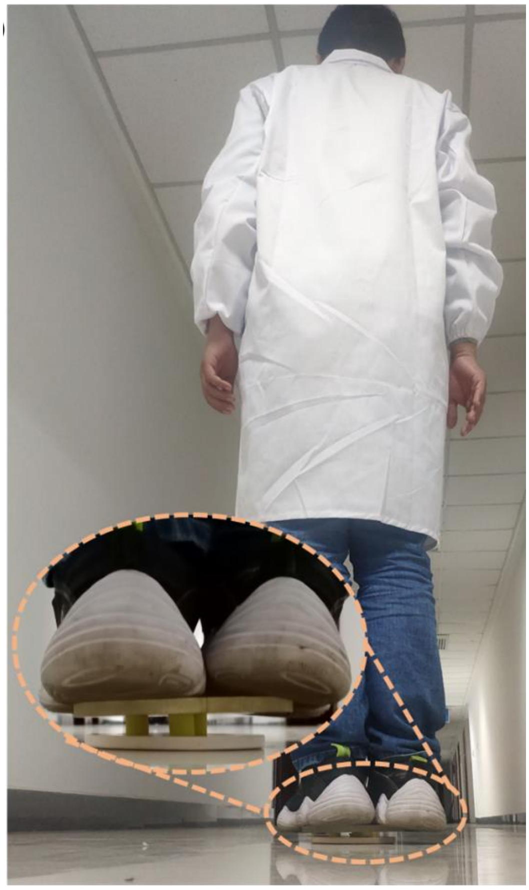

Figure S3. Diagram of aerogel carrying adults.

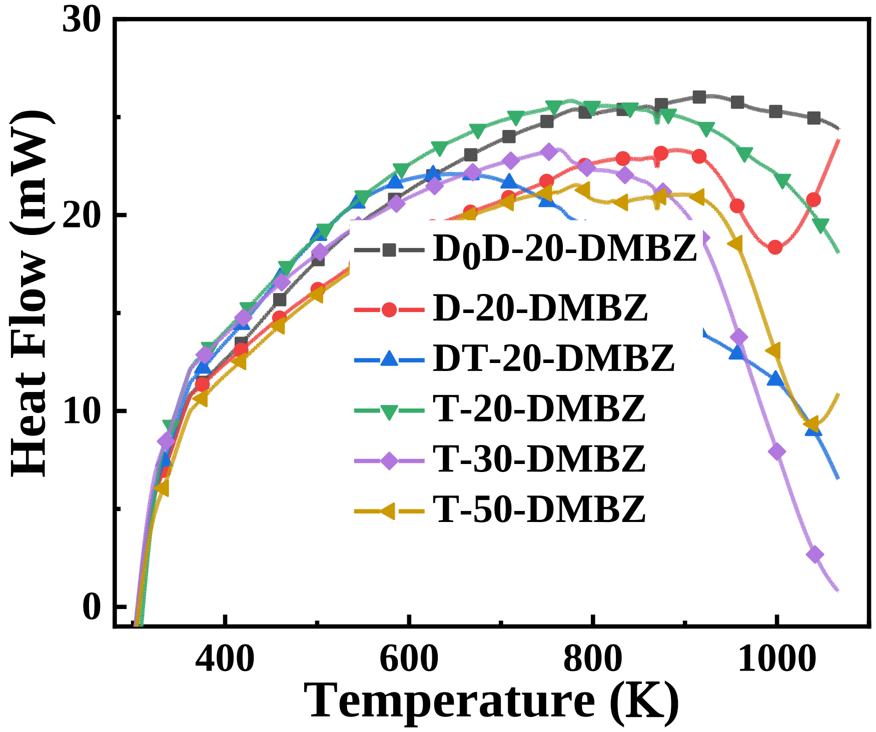

Figure S4. The heat flow curves of aerogels cross-linked with silicone polymers prepared from different precursors.
